# Estimated Acute Effects of Ambient Ozone and Nitrogen Dioxide on Mortality in the Pearl River Delta of Southern China

**DOI:** 10.1289/ehp.1103715

**Published:** 2011-12-08

**Authors:** Yebin Tao, Wei Huang, Xiaoliang Huang, Liuju Zhong, Shou-En Lu, Yi Li, Lingzhen Dai, Yuanhang Zhang, Tong Zhu

**Affiliations:** 1 SKJ Laboratory of Environmental Simulation and Pollution Control, College of Environmental Sciences and Engineering, and Centre for Environment and Health, Peking University, Beijing, China; 2 Consensus Information Center, Guangdong Provincial Bureau of Hygiene, Guangzhou, China; 3 Guangdong Provincial Environmental Monitoring Center, Guangzhou, China; 4 Department of Biostatistics, University of Medicine and Dentistry of New Jersey, Piscataway, New Jersey, USA; 5 Chinese Academy of Meteorological Sciences, Beijing, China

**Keywords:** excess risk, mortality, nitrogen dioxide, ozone, PRD, time series

## Abstract

Background and objectives: Epidemiologic studies have attributed adverse health effects to air pollution; however, controversy remains regarding the relationship between ambient oxidants [ozone (O_3_) and nitrogen dioxide (NO_2_)] and mortality, especially in Asia. We conducted a four-city time-series study to investigate acute effects of O_3_ and NO_2_ in the Pearl River Delta (PRD) of southern China, using data from 2006 through 2008.

Methods: We used generalized linear models with Poisson regression incorporating natural spline functions to analyze acute mortality in association with O_3_ and NO_2_, with PM_10_ (particulate matter ≤ 10 μm in diameter) included as a major confounder. Effect estimates were determined for individual cities and for the four cities as a whole. We stratified the analysis according to high- and low- exposure periods for O_3_.

Results: We found consistent positive associations between ambient oxidants and daily mortality across the PRD cities. Overall, 10-μg/m^3^ increases in average O_3_ and NO_2_ concentrations over the previous 2 days were associated with 0.81% [95% confidence interval (CI): 0.63%, 1.00%] and 1.95% (95% CI: 1.62%, 2.29%) increases in total mortality, respectively, with stronger estimated effects for cardiovascular and respiratory mortality. After adjusting for PM_10_, estimated effects of O_3_ on total and cardiovascular mortality were stronger for exposure during high-exposure months (September through November), whereas respiratory mortality was associated with O_3_ exposure during nonpeak exposure months only.

Conclusions: Our findings suggest significant acute mortality effects of O_3_ and NO_2_ in the PRD and strengthen the rationale for further limiting the ambient pollution levels in the area.

Substantial evidence supports the association between ambient air pollution, including particulate matter (PM), ozone (O_3_), and nitrogen dioxide (NO_2_), and mortality and morbidity from cardiopulmonary diseases (Bell et al. 2004; Brook et al. 2010; Dockery et al. 1993; Pope and Dockery 2006; Pope et al. 2002; Samet et al. 2000). However, although higher concentrations of air pollution are often found in Asia, published Asian research is still limited, making region-specific results difficult to interpret and compare with findings for populations in the developed world (Cao et al. 2011; Chen et al. 2004; Wong et al. 2008; Zhou et al. 2010).

Chinese megacities are some of the most air-polluted cities in the world (Chan and Yao 2008). There has been growing concern about air-pollution–related health effects in the Pearl River Delta (PRD), which recently has undergone rapid economic development and urbanization. The PRD lies in the coastal part of southern China and comprises nine cities in Guangdong Province and the special administrative regions of Hong Kong and Macao. This region accounts for only 0.5% of the Chinese geographic area but holds 4% of the Chinese population and produces about one-fifth of the total gross domestic product. Remarkable problems of traffic and photochemical air pollution have emerged in the PRD, in large part due to vehicle emissions and high sun exposure year-round (Shao et al. 2009). Photochemical reactions involving nitrogen oxides, volatile organic compounds, and hydroxyl radicals produce O_3_, a secondary air pollutant, in the presence of sunlight [Hofzumahaus et al. 2009; World Health Organization (WHO) 2005]. The PRD was one of the first regions in China to experience serious photochemical smog pollution: up to 171 ppb O_3_ was measured in a suburb of Hong Kong, with sizable contributions likely from air mass transported from Guangdong cities in the PRD (So and Wang 2003). The PRD Regional Air Quality Monitoring Network, with 16 monitoring stations across the region, was established to facilitate research on pollution in the PRD (Hua et al. 2008; Zhang et al. 2008a).

Ambient O_3_ and NO_2_, two major oxidants involved in photochemical processes, have been associated with adverse health effects. A systematic review indicates that the reaction between O_3_ and biomolecules to form ozonides and free radicals triggers inflammatory responses and systemic oxidative stress in the cardiorespiratory system (Srebot et al. 2009). NO_2_ is a highly reactive and nitrogen-centered free radical that can induce airway inflammation (Kelly et al. 1996). Short-term exposure to ambient O_3_ and NO_2_ has adverse effects on pulmonary function, particularly in asthmatics, and may increase airway allergic inflammatory reactions, hospital admissions, and mortality (WHO 2004). Many epidemiologic studies suggested both short- and long-term effects of exposure to O_3_ (Bell et al. 2004, 2005; Gryparis et al. 2004; Jerrett et al. 2005) and NO_2_ (Hoek et al. 2002; Samoli et al. 2006) on total and cause-specific mortality. A few studies focused on both oxidants as reactive components of the photochemical air pollution mixture and reported significant mortality effects of short-term oxidant exposure (Saez et al. 2002; Touloumi et al. 1997).

Air monitoring data collected through the PRD monitoring network have made it possible to assess the health effects of ambient air pollution on a regional scale. In this multicity study, we estimated associations between ambient O_3_ and NO_2_ and mortality in the PRD (2006–2008), using a time-series approach.

## Materials and Methods

**Figure 1:**
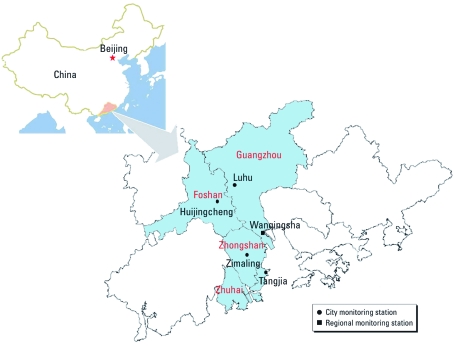
The PRD and locations of air pollution monitors in the four cities included in the study: Guangzhou, Foshan, Zhongshan, and Zhuhai.

*Study site description.* Our study cities included Guangzhou, Foshan, Zhongshan, and Zhuhai in the PRD (Figure 1). Guangzhou, the capital city of Guangdong Province, is a megacity that had 7.8 million urban residents in 2008; Foshan, Zhongshan, and Zhuhai had 3.8, 1.5, and 1.0 million residents, respectively. These cities have a typical monsoon-influenced climate with wet, hot summers and dry, cool to mild winters. The annual average precipitation is approximately 1,800 mm, the annual average temperature is 22–23°C, and the annual average relative humidity (RH) is 72–80%. The predominant southern or southeastern winds from the South China Sea during spring and summer bring relatively clean oceanic air, whereas northeastern winds carry air pollutants from close-vicinity northern cities in autumn and winter. These cities are typical of the PRD cities with respect to geographical, meteorological, and cultural conditions, although they vary in size and industrial structure.

*Mortality data.* We obtained daily mortality data for 2006 through 2008 on total (nonaccidental) deaths [*International Classification of Diseases*, *10th Revision* (ICD-10; WHO 1994), codes A00–R99] and deaths attributed to cardiovascular diseases (codes I00–I99) and respiratory diseases (codes J00–J98) at all ages. The data also included subcategories of cardiovascular and respiratory mortality: coronary (codes I00–I09 and I20–I52), stroke (codes I60–I69), and chronic obstructive pulmonary disease (COPD; codes J40–J47).

*Environmental data.* Air pollution data were measured at five monitoring stations in the area: two in Guangzhou (one regional monitoring station in Wanqingsha and one in the central city park Luhu), and one station each in Foshan, Zhongshan, and Zhuhai (Figure 1). All monitoring stations except the Luhu station are in urban areas with mixed residential and commercial activities. The monitors sample air about 10–20 m above ground level.

We obtained hourly concentrations of NO_2_, O_3_, sulfur dioxide (SO_2_), carbon monoxide (CO), and PM_10_ (PM with aerodynamic diameter ≤ 10 μm) from each station. NO_2_, SO_2_, O_3_, and CO were measured using chemiluminescence, fluorescence, ultraviolet, and infrared instruments, respectively; PM_10_ was measured by tapered element oscillating microbalance. We calculated 24-hr average concentrations for PM_10_, NO_2_, SO_2_, and CO for days that had measurements for at least 18 of 24 hr. We calculated 8-hr (from 1000 hours to 1800 hours) average concentrations for O_3_ for days with at least six of eight hourly measurements available. If monitor data for an individual pollutant were insufficient to calculate a daily average, all measurements from that day were excluded for that pollutant and monitor. Data from the two monitoring stations were averaged to derive concentrations for Guangzhou. Daily temperature and RH data for each city were obtained from the Chinese Academy of Meteorological Sciences. Missing data were not imputed.

*Statistical analysis.* Because daily mortality counts typically follow a Poisson distribution, we used Poisson regression models to evaluate the association between mortality and air pollution controlling for temperature, RH, seasonal patterns, and long-term trends using natural spline smoothers (Burnett et al. 2004; Samet and Katsouyanni 2006; Wood 2006). Degrees of freedom of the natural spline smoothers were determined by Akaike’s information criterion (Hurvich et al. 1998) and generalized cross-validation. If there was overdispersion in the variance, we used the partial autocorrelation function (PACF) of the residuals to guide the selection of degrees of freedom until the absolute values of sum of PACF for lags up to 30 days reached minimum. Analyses were also adjusted for year, day of the week (DOW), and public holidays using categorical indicator variables. We adjusted for influenza epidemics by including an indicator variable that was assigned a value of 1 when the 7-day moving average of the respiratory mortality was greater than the 90th percentile of its city-specific distribution, and 0 otherwise (Samoli et al. 2006). Because the influenza variable was based on the distribution of respiratory mortality, and because previous studies (Braga et al. 2000; Touloumi et al. 2005) suggest that omitting control for influenza is unlikely to influence the association between air pollution and respiratory mortality, we adjusted for influenza only in models of total mortality and cardiovascular mortality (Touloumi et al. 2004). Residuals of each model were examined for discernible patterns and autocorrelation using residual plots and PACF plots, respectively [Health Effects Institute (HEI) 2010b].

Associations between mortality and average air pollutant concentrations on individual days (lag 0 to lag 6) and 2-day periods (lag 0–1 days and lag 1–2 days) were first examined in single-pollutant models. Preliminary analyses indicated that the largest pollutant effects were usually observed at lag 1–2 days (data not shown). Therefore, we report the excess risk (ER) of mortality and its 95% confidence interval (CI) associated with a 10-μg/m^3^ increase in the average concentration of each pollutant during the previous 1–2 days. Single-day temperature and RH (lag 0 or lag 1 day) were used in our analyses, and the reported results were from models with lag 1 day covariates.

After establishing the final model that controlled for time trend, temperature, RH, year, DOW, public holiday, and influenza epidemics, we calculated city-specific estimates by fitting Poisson regression models for each city separately. We calculated *Q*-statistics to test the homogeneity of effect estimates among the study cities (α = 0.05) (DerSimonian and Laird 1986). Because the cities are all located along the estuary of the Pearl River, in close proximity to each other (Figure 1), and are similar with regard to natural and social factors, and because measurements from the five monitoring stations were correlated (Pearson correlation coefficients ranged from 0.64 to 0.88 for O_3_ and from 0.43 to 0.83 for NO_2_), we also generated combined effect estimates by summing the mortality data across the four cities and averaging the environmental data from the five monitoring stations.

In the city-merged analyses, both single- and two-pollutant models were applied to estimate the effects of O_3_, NO_2_, and PM_10_ adjusted for confounding by other pollutants. Two-pollutant models were restricted to pollutants with Pearson correlation coefficients < 0.6 to avoid multicollinearity. Furthermore, we stratified O_3_ exposure as exposure during peak (September through November) and nonpeak (December through August) exposure periods identified previously for the area (Zhang et al. 2008b; Zheng et al. 2010) by using 2-df splines to control for time trend during the peak period and 6-df splines for the nonpeak period (Zanobetti and Schwartz 2008). In the stratified analysis, we adjusted for PM_10_, because stratum-specific effects of O_3_ could be confounded by PM_10_ (Bell et al. 2007).

Finally, we conducted a series of sensitivity analyses focused on O_3_ to assess the impact of dropping model covariates, increasing or decreasing the degrees of freedom for time and meteorological spline variables by 25%, lagging temperature by 2–3 days or 4–6 days, and excluding days with daily concentrations of O_3_ above the 95th or below the 5th percentile.

Analyses were performed using R (version 2.13.0; R Foundation for Statistical Computing, Vienna, Austria) with the mgcv package (version 1.5–5; Comprehensive R Archive Network, http://cran.r-project.org). Statistical significance was defined as *p* < 0.05.

## Results

Table 1 summarizes the mortality data for the four PRD cities from 2006 through 2008. Average daily nonaccidental death counts were 83, 25, 21, and 9 for Guangzhou, Foshan, Zhongshan, and Zhuhai, respectively. About 56% of all nonaccidental deaths occurred in males, and 54–63% were attributed to cardiovascular or respiratory diseases.

**Table 1:** Summary statistics of daily mortality counts in the PRD cities, 2006–2008 [mean ± SD (range)].

Mortality	Guangzhou	Foshan	Zhongshan	Zhuhai
Total								
Nonaccidental		83.2 ± 16.5 (47–173)		24.5 ± 8.2 (0–104)		20.7 ± 6.1 (5–52)		8.5 ± 3.2 (1–22)
Female		36.5 ± 9.2 (15–87)		10.9 ± 4.5 (0–45)		9.1 ± 3.6 (1–25)		3.7 ± 2.1 (0–12)
Male		46.7 ± 9.8 (20–94)		13.6 ± 5.2 (0–59)		11.6 ± 4.0 (1–27)		4.8 ± 2.3 (0–13)
Cardiovascular		30.1 ± 8.5 (11–74)		9.3 ± 4.4 (0–45)		9.4 ± 3.8 (0–26)		3.5 ± 2.2 (0–15)
Coronary		15.6 ± 5.2 (2–39)		5.0 ± 2.7 (0–25)		4.4 ± 2.3 (0–17)		1.4 ± 1.3 (0–8)
Stroke		10.6 ± 4.0 (1–28)		3.1 ± 2.3 (0–15)		2.2 ± 1.6 (0–9)		1.7 ± 1.4 (0–8)
Respiratory		15.2 ± 5.2 (5–44)		5.3 ± 3.0 (0–25)		3.7 ± 2.2 (0–13)		1.1 ± 1.1 (0–6)
COPD		7.7 ± 3.5 (0–27)		2.2 ± 1.6 (0–11)		2.3 ± 1.7 (0–11)		0.8 ± 0.9 (0–5)


**Figure 2:**
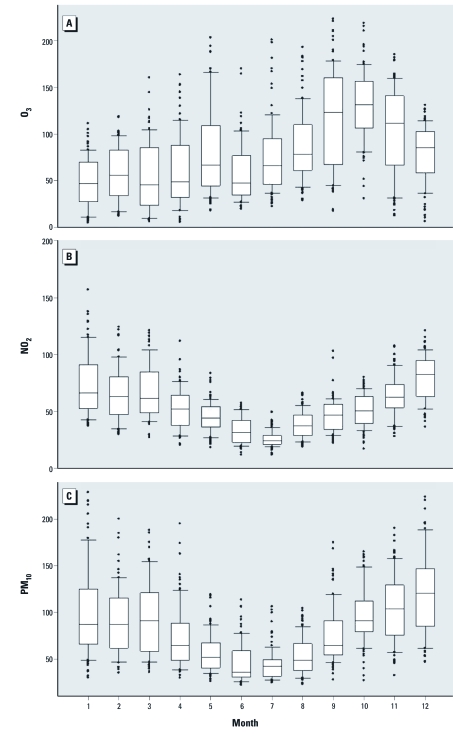
Box plots of monthly O_3_, NO_2_, and PM_10_ concentrations (μg/m^3^): averaged data from the five monitoring stations, 2006–2008. Boxes indicate the interquartile range (25th to 75th percentile); lines within boxes indicate medians; whiskers represent 5th and 95th percentile values; and circles represent outliers.

Table 2 presents the air pollution levels of the four cities from 2006 through 2008. Foshan had the highest average concentrations of PM_10_, NO_2_, SO_2_, and CO, and Zhongshan had the highest average concentration of O_3_. Monthly O_3_ concentrations were highest during the peak exposure period from September through November (Figure 2). Based on combined data for the four cities, O_3_ was the least correlated with other pollutants (Pearson correlation coefficients, –0.06 to 0.17), whereas NO_2_ was highly correlated with PM_10_, SO_2_, and CO (correlation coefficients, 0.72–0.82). Temperature was positively correlated with O_3_ only, whereas RH was negatively correlated with all the pollutants (Table 3).

**Table 2:** Summary statistics of ambient air pollutant concentrations in the PRD cities, 2006–2008 [mean (interquartile range)].

Pollutant	Guangzhou	Foshan	Zhongshan	Zhuhai
PM_10_ (μg/m^3^)		81.0 (62.0)		121.3 (89.0)		64.2 (58.2)		43.5 (24.8)
NO_2_ (μg/m^3^)		53.9 (33.1)		70.4 (39.1)		48.4 (42.4)		38.1 (34.2)
O_3_ (μg/m^3^)		78.2 (72.8)		70.7 (77.3)		85.7 (70.8)		85.5 (76.5)
SO_2_ (μg/m^3^)		55.2 (35.2)		95.4 (73.2)		57.3 (72.1)		39.5 (40.0)
CO (ppm)		1.35 (0.61)		1.65 (1.23)		1.20 (0.87)		1.29 (0.73)
Data are 24-hr averages for PM_10_, NO_2_, SO_2_, and CO and 8-hr (1000 hours to 1800 hours) averages for O_3_.								

**Table 3:** Pearson correlation coefficients between daily air pollutant concentrations, temperature, and RH in the PRD, using city-merged data, 2006–2008.

Variable	NO_2_	PM_10_	SO_2_	CO	Temperature	RH
O_3_		0.17		0.36		0.08		–0.06		0.30		–0.50
NO_2_		1.00		0.82		0.82		0.72		–0.51		–0.31
PM_10_				1.00		0.71		0.64		–0.34		–0.41
SO_2_						1.00		0.74		–0.47		–0.31
CO								1.00		–0.52		–0.09
Temperature										1.00		0.29


In the individual cities, adjusted ERs of total mortality in association with a 10-μg/m^3^ increase in exposure (1–2 day lag, single-pollutant models) ranged from 0.22% to 0.64% for O_3_, 1.22% to 1.87% for NO_2_, and 0.37% to 0.74% for PM_10_ (Table 4). In general, we observed stronger associations of O_3_, NO_2_, and PM_10_ with cardiovascular and respiratory mortality than with total mortality. Although there was heterogeneity for the associations between PM_10_ and respiratory mortality and also some variation in Foshan and Zhuhai, no significant heterogeneity was found in the effect estimates of O_3_ and NO_2_ among the four cities, which supported our analyses with city-merged data.

**Table 4:** ER of mortality associated with a 10-μg/m^3^ increase in lag 1–2 day O_3_, NO_2_, and PM_10_ concentrations in individual cities based on single-pollutant models [percent (95% CI)].*^a^*

Megacity: Guangzhou	Medium-sized cities					*p*-Value for homogeneity test
	Mortality	Pollutant	Foshan	Zhongshan	Zhuhai	
Total (nonaccidental)		O_3_		0.64 (0.42, 0.86)		0.36 (–0.06, 0.78)		0.61 (0.22, 1.00)		0.22 (–0.36, 0.81)		0.432
		NO_2_		1.66 (1.28, 2.05)		1.87 (1.40, 2.35)		1.22 (0.44, 2.01)		1.39 (–0.09, 2.89)		0.555
		PM_10_		0.74 (0.53, 0.95)		0.50 (0.31, 0.69)		0.44 (–0.02, 0.91)		0.37 (–0.97, 1.73)		0.351
Cardiovascular		O_3_		0.98 (0.61, 1.35)		0.43 (–0.25, 1.12)		0.77 (0.19, 1.35)		–0.08 (–1.00, 0.85)		0.144
		NO_2_		1.92 (1.29, 2.57)		2.35 (1.59, 3.13)		1.19 (0.05, 2.34)		2.22 (–0.09, 4.58)		0.426
		PM_10_		0.92 (0.58, 1.26)		0.71 (0.40, 1.01)		0.45 (–0.23, 1.14)		0.47 (–1.62, 2.60)		0.611
Respiratory		O_3_		0.89 (0.38, 1.41)		0.46 (–0.43, 1.36)		0.61 (–0.32, 1.55)		1.61 (–0.05, 3.30)		0.631
		NO_2_		2.99 (2.13, 3.86)		1.60 (0.60, 2.61)		3.44 (1.67, 5.25)		2.46 (–1.59, 6.67)		0.147
		PM_10_		1.20 (0.72, 1.68)		0.09 (–0.32, 0.50)		1.00 (–0.09, 2.10)		2.82 (–0.96, 6.75)		0.003
**a**Poisson regression model controlled for time trend, temperature, RH, year, DOW, public holiday, and influenza epidemics.												

In the city-merged analyses, effect estimates for O_3_ were moderately reduced but still significant after adjustment for PM_10_, NO_2_, SO_2_, and CO. Effect estimates for NO_2_ and PM_10_ were also attenuated but still significant after adjustment for O_3_ (Table 5). The variation of effect estimates among pollutants became smaller when assessment was made per interquartile range (IQR) increase. For instance, the effect estimates of O_3_ and NO_2_ on total mortality were 0.81% and 1.95% per 10-μg/m^3^ increase for each pollutant (Table 5), whereas the estimates were 5.31% and 5.97% per IQR increase [see Supplemental Material, Table 1 (http://dx.doi.org/10.1289/ehp.1103715)]. We also observed significant increased risk in cardiorespiratory mortality per unit increase in air pollution: a 10-μg/m^3^ increase in O_3_ exposure was associated with 0.79% (95% CI: 0.36, 1.22%), 1.17% (95% CI: 0.65, 1.70%), and 1.16% (95% CI: 0.56, 1.77%) increases in mortality from coronary, stroke, and COPD diseases, respectively (see Supplemental Material, Table 2).

**Table 5:** ER of nonaccidental mortality associated with a 10-μg/m^3^ increase in lag 1–2 day O_3_, NO_2_, and PM_10_ concentrations under single- and two-pollutant models*a* based on combined data for four cities in the PRD [percent (95% CI)].

Pollutant	Method	Total	Cardiovascular	Respiratory
O_3_		Single-pollutant model		0.81 (0.63, 1.00)		1.01 (0.71, 1.32)		1.33 (0.89, 1.76)
		Adjusted for PM_10_		0.54 (0.34, 0.75)		0.71 (0.37, 1.05)		0.87 (0.39, 1.36)
		Adjusted for NO_2_		0.43 (0.23, 0.64)		0.62 (0.29, 0.96)		0.58 (0.10, 1.06)
		Adjusted for SO_2_		0.70 (0.51, 0.90)		0.92 (0.60, 1.23)		1.16 (0.71, 1.61)
		Adjusted for CO		0.72 (0.53, 0.91)		0.89 (0.58, 1.20)		1.17 (0.72, 1.61)
NO_2_		Single-pollutant model		1.95 (1.62, 2.29)		2.12 (1.58, 2.65)		3.48 (2.73, 4.23)
		Adjusted for O_3_		1.63 (1.27, 2.00)		1.67 (1.08, 2.25)		3.07 (2.25, 3.89)
PM_10_		Single-pollutant model		0.79 (0.62, 0.96)		0.91 (0.64, 1.19)		1.26 (0.88, 1.65)
		Adjusted for O_3_		0.58 (0.39, 0.76)		0.64 (0.34, 0.95)		0.93 (0.51, 1.36)
**a**Two-pollutant models were limited to pollutants with Pearson correlation coefficients < 0.6; other covariates controlled for were the same as those in Table 4.								

Average concentrations of O_3_ were 117.4 μg/m^3^ in the peak exposure period (September through November) and 66.9 μg/m^3^ in the nonpeak period. O_3_ exposure was significantly associated with total mortality and cardiovascular mortality in both periods (Table 6). After adjustment for PM_10_, effect estimates for total and cardiovascular mortality increased in the peak period but decreased in the nonpeak period. Respiratory mortality was significantly associated with O_3_ exposure during the nonpeak period only, and there was no evidence of an association during the peak period after adjusting for PM_10_.

**Table 6:** ER of mortality associated with a 10-μg/m^3^ increase in lag 1–2 day O_3_ concentrations by O_3_ exposure period,*a* under single- and two-pollutant models, using city-merged data [percent (95% CI)].

Mortality	Method	Peak period	Nonpeak period
Total		Single-pollutant model		0.65 (0.27, 1.02)		0.92 (0.69, 1.16)
		Adjusted by PM_10_		0.77 (0.32, 1.22)		0.64 (0.39, 0.89)
Cardiovascular		Single-pollutant model		0.96 (0.35, 1.58)		1.06 (0.69, 1.44)
		Adjusted by PM_10_		1.33 (0.59, 2.08)		0.67 (0.27, 1.07)
Respiratory		Single-pollutant model		0.24 (–0.63, 1.13)		2.00 (1.47, 2.53)*
		Adjusted by PM_10_		0.08 (–0.98, 1.16)		1.62 (1.05, 2.20)*
**a**Peak period (September through November) with 8-hr mean O_3_ of 117.4 μg/m^3^ and nonpeak period (December through August) with 8-hr mean O_3_ of 66.9 μg/m^3^. Other covariates controlled for were the same as those in Table 4. *Significantly different from the peak period (*p* < 0.01).						

Our sensitivity analyses indicated that covariates did not introduce collinearity, and all were significant predictors (*p* < 0.05). Altering the degrees of freedom of time and meteorological smoothers and excluding days with extremely high or low O_3_ concentrations did not alter total mortality effect estimates by > 20% [see Supplemental Material, Table 3 (http://dx.doi.org/10.1289/ehp.1103715)]. However, estimates for total mortality were somewhat sensitive to adjustment for temperature over different lag periods, resulting in ERs for a 10-μg/m^3^ increase in O_3_ (1- to 2-day lag) of 0.70% (95% CI: 0.51%, 0.89%) and 0.63% (95% CI: 0.44%, 0.82%) when adjusted for temperature with a lag of 2–3 days or 4–6 days, respectively, compared with 0.81% (95% CI: 0.63%, 1.00%) when adjusted for temperature with a 1-day lag.

## Discussion

We estimated significant acute mortality effects associated with exposure to ambient oxidants in the PRD. In general, associations between O_3_ and NO_2_ exposure and daily mortality were homogeneous among the study cities. The estimated effects of O_3_ were robust to adjustment for other pollutants (PM_10_, NO_2_, SO_2_, CO), and effect estimates for NO_2_ were robust to adjustment for O_3_. Effect estimates for O_3_ were larger for exposure during the peak period for total and cardiovascular mortality after adjustment for PM_10_, whereas for respiratory mortality, the association appeared to be limited to exposure during the nonpeak period.

We observed much higher concentrations of O_3_ in the PRD cities (annual mean, 70–85 μg/m^3^) than those observed in North American cities (14–38 μg/m^3^) (Samet and Katsouyanni 2006). Our analysis indicated significant increases of 0.81% (95% CI: 0.63%, 1.00%) in total mortality, 1.01% (95% CI: 0.71%, 1.32%) in cardiovascular mortality, and 1.33% (95% CI: 0.89%, 1.76%) in respiratory mortality, per 10-μg/m^3^ increase in lag 1–2 day O_3_ level in the PRD. Consistently, a multisite time-series study of 95 large U.S. urban communities estimated that a 10-ppb (≈ 20 μg/m^3^) increase in the previous week’s O_3_ was associated with a 0.52% (95% CI: 0.27%, 0.77%) increase in daily mortality and a 0.64% (95% CI: 0.31%, 0.98%) increase in cardiovascular and respiratory mortality (Bell et al. 2004). A meta-analysis of 144 effect estimates from 39 time-series studies also provided strong evidence of a short-term association between O_3_ exposure and mortality, with larger estimated effects on cardiovascular and respiratory mortality than on total mortality (Bell et al. 2005). However, several other studies reported acute O_3_ exposure effects on cardiovascular mortality but not respiratory mortality, including an analysis of seven Spanish cities in the EMECAM (Spanish Multicenter Study on the Relationship between Air Pollution and Mortality) project (Saez et al. 2002) and studies in Asia (HEI 2010b; Zhang et al. 2006). Wong et al. (2008) reported that effect estimates of O_3_ were significant for total and cardiovascular mortality but only marginally significant for respiratory mortality in four Asian cities. In addition, a recent review of Asian studies reported positive but much smaller and inconsistent effect estimates for O_3_ and mortality across cities (HEI 2010a). Considering the limited number of estimates available for meta-analysis, future studies should be conducted in more Asian cities in order to address the inconsistency.

Consistent with previous studies (Burnett et al. 2004; Michelozzi et al. 1998; Touloumi et al. 1997), our analysis indicated significant associations between short-term change in NO_2_ and mortality in the PRD, with estimated increases of 1.95% (95% CI: 1.62%, 2.29%) in total mortality, 2.12% (95% CI: 1.58%, 2.65%) in cardiovascular mortality, and 3.48% (95% CI: 2.73%, 4.23%) in respiratory mortality, per 10-μg/m^3^ increase in lag 1–2 day NO_2_ concentrations. However, the magnitude of NO_2_ effect estimates in our study was much greater than comparable estimates reported for Western populations (pooled estimates, 0.30–0.50% increases in total, cardiovascular, and respiratory mortality per 10-μg/m^3^ increase in lag 0–1 day NO_2_ concentrations) (Samoli et al. 2006). Our estimates were also larger than comparable estimates for other Asian cities (HEI 2010b) and summary estimates of 0.98% (95% CI: 0.54%, 1.42%), 1.74% (95% CI: 0.85%, 2.63%), and 1.08% (95% CI: 0.59%, 1.56%) for total, cardiovascular, and respiratory mortality per 10-μg/m^3^ increase in NO_2_ based on a meta-analysis of Asian time-series studies (HEI 2010a).

Ambient oxidants of O_3_ and NO_2_ are major air pollutants in the PRD. Estimated effects of O_3_ were moderately reduced but still significant after adjustment for PM_10_, SO_2_, and CO in two-pollutant models. This is probably because O_3_, as a secondary pollutant, has formation paths in the environment different from those of PM_10_, SO_2_, and CO and thus does not typically covary with these pollutants. With weak correlations observed between O_3_ and other pollutants, the mortality effect of O_3_ exposure is at least partially independent of other pollutants, which is consistent with findings reported previously (Bell et al. 2007; Gryparis et al. 2004). Unlike O_3_, NO_2_ could be a proxy marker of exposure to pollutants (including PM_10_, SO_2_, and CO) generated by the same sources, such as vehicle emissions, or may reflect the combined effects of pollutants related to traffic and atmospheric photochemical pollution in the PRD. The significant associations estimated for O_3_ and NO_2_ suggest that ambient oxidants may play important roles in initiating air pollution-related mortality effects in the PRD.

A major finding of our study was the differential mortality effects of O_3_ estimated for exposures during the peak and nonpeak exposure periods. The PRD lies on the border between subtropical and tropical zones, and the highest O_3_ concentrations often occur in autumn (September through November), in contrast with other geographic areas studied, where peak O_3_ period occurs in summer (June through August), (Bell et al. 2005; Zanobetti and Schwartz 2008). Correlation analysis indicated that O_3_ concentrations were positively correlated with temperature and negatively correlated with RH in the area. Although the formation of O_3_ relies largely on sunlight, the diffusion and clearance of O_3_ can be accelerated by precipitation in summer. Therefore, peak levels of O_3_ in the PRD are often observed in autumn, when it is drier than in the summer (Shao et al. 2009). In the stratified analysis of O_3_ after adjustment for PM_10_, we observed higher risk of O_3_ exposure for total and cardiovascular mortality in the peak period, which was consistent with many Western studies that reported significant associations in summer or warm months (April through September) when the O_3_ level is higher (Bell et al. 2005; Gryparis et al. 2004; Ito et al. 2005; Schwartz 2005). Although the significantly higher association observed between O_3_ and respiratory mortality in the nonpeak exposure period differed from those observed in Western studies, similar results have been reported for Hong Kong, where associations between respiratory mortality and O_3_ exposure were stronger in cold seasons with lower O_3_ levels (Wong et al. 1999, 2001). People with respiratory diseases may be more sensitive to high O_3_ exposure than are people with other diseases; thus, with very high mean level of 117.4 μg/m^3^ measured in the peak period, the risks of respiratory mortality could be reduced because vulnerable subjects may have died before the O_3_ concentration reached higher levels (Wong et al. 2001).

Limitations should be noted in interpreting the results of our study. First, exposure data were obtained from only one or two air pollution monitoring stations in each city. Air pollution exposure may be spatially autocorrelated across our study cities, which may violate the assumption of independent exposures among cities for the merged regression analyses. The limited number of air pollution monitoring stations and exposure data to assess spatial structure of air pollution between cities may yield biased variance of parameter estimates and inefficient significance tests in our study (Jerrett et al. 2005). Second, no PM_2.5_ and component data were available for the study period. Compared with PM_10_, PM_2.5_ is a better index of combustion source air pollution and has a larger proportion of secondary particles. Franklin and Schwartz (2008) suggested that some secondary particle, such as particulate sulfate, may be partly responsible for observed O_3_ effects. Therefore, adjusting for PM_10_, instead of PM_2.5_ or its components, may overestimate the effect of O_3_.

## Conclusion

We estimated significant increases in mortality associated with O_3_ and NO_2_ exposures in the PRD. The evidence of differential effects of O_3_ on mortality from different diseases supports the need for further investigation of the pathophysiological mechanisms of O_3_-associated cardiovascular and respiratory effects. Our findings strengthen the rationale for further limiting ambient oxidant pollution in the PRD.

## Supplemental Material

(94 KB) PDF
