# Supplementary material for: Estimated Acute Effects of Ambient Ozone and Nitrogen Dioxide on Mortality in the Pearl River Delta of Southern China
Source: Environ Health Perspect. 2011 Dec 8;120(3):393–8. doi: 10.1289/ehp.1103715 (PMC3295344; doi:10.1289/ehp.1103715)
Supplement: (94 KB) PDF [file ehp.1103715.s001.pdf]

Supplemental Material

Estimated Acute Effects of Ambient Ozone and Nitrogen Dioxide on Mortality in the Pearl River Delta of Southern China

Yebin Tao, Wei Huang, Xiaoliang Huang, Liuju Zhong, Shou-En Lu, Yi Li, Lingzhen Dai, Yuanhang Zhang, Tong Zhu

Table of Contents:

Supplemental Material, Table 1. Effect estimates (%, 95% CI) of O_3, NO_2 and PM_{10} on total, cardiovascular and respiratory mortality per IQR increase in lag 1-2 day concentrations, using city-merged data.

Supplemental Material, Table 2. Effect estimates (%, 95% CI) of O_3, NO_2 and PM_{10} on subcategories of cardiovascular and respiratory mortality per 10μg/m^{3}increase in lag 1-2 day concentrations, using city-merged data.

Supplemental Material, Table 3.Excess risk (%, 95% CI) of mortality for a 10-μg/m^3 increase in lag 1-2 day O_3 concentrations by sensitivity analyses with variation in methods and concentration levels.

Supplemental Material, Table 1. Effect estimates (%, 95% CI) of O_3, NO_2 and PM_{10} on total, cardiovascular and respiratory mortality per IQR[a] increase in lag 1-2 day concentrations, using city-merged data.

Mortality	O_3	NO_2	PM_{10}
Total	5.31 (4.06 to 6.56)	5.97 (4.93 to 7.02)	4.42 (3.45 to 5.40)
Cardiovascular	6.65 (4.62 to 8.72)	6.48 (4.81 to 8.18)	5.14 (3.58 to 6.73)
Respiratory	8.77 (5.82 to 11.81)	10.80 (8.41 to 13.24)	7.18 (4.95 to 9.46)

Note: [a]IQR (μg/m^3): 63.8 for O_3, 30.8 for $NO_2$and 55.2 for PM_{10} at average lag 1-2 days; Poisson regression model controlled for time trend, temperature, RH, year, DOW, public holiday and influenza epidemics.

Supplemental Material, Table 2.Effect estimates (%, 95% CI) of O_3, NO_2 and PM_{10} on subcategories of cardiovascular and respiratory mortality per 10μg/m^3increase in lag 1-2 day concentrations, using city-merged data.

Mortality	O_3	NO_2	PM_{10}
Cardiovascular	1.01 (0.71 to 1.32)	2.12 (1.58 to 2.65)	0.91 (0.64 to 1.19)
Coronary	0.79 (0.36 to 1.22)	1.79 (1.04 to 2.55)	0.93 (0.54 to 1.31)
Stroke	1.17 (0.65 to 1.70)	2.58 (1.66 to 3.51)	1.02 (0.55 to 1.49)
Respiratory	1.33 (0.89 to 1.76)	3.48 (2.73 to 4.23)	1.26 (0.88 to 1.65)
COPD	1.16 (0.56 to 1.77)	2.97 (1.95 to 4.00)	1.32 (0.79 to 1.86)

Note: Poisson regression model controlled for time trend, temperature, RH, year, DOW, public holiday and influenza epidemics.

Supplemental Material, Table 3. Excess risk (%, 95% CI) of mortality per 10-μg/m^3 increase in lag 1-2 day O_3 concentrations by sensitivity analyses with variation in methods and concentration levels.

Total mortality	ER	95% CI
Main analysis	0.81	0.63 to 1.00
Add temperature at lag 2-3 days	0.70	0.51 to 0.89
Add temperature at lag 4-6 days	0.63[a]	0.44 to 0.82
- 25% df for time smoothing	0.75	0.57 to 0.94
+ 25% df for time smoothing	0.85	0.66 to 1.04
- 25% df for meteorological smoothing	0.88	0.70 to 1.06
+ 25% df for meteorological smoothing	0.81	0.62 to 1.01
Omit O_3> 95th percentile (166.6 μg/m^3)	0.82	0.60 to 1.04
Omit O_3< 5th percentile (18.3μg/m^3)	0.78	0.59 to 0.98

Note: [a]ER changed > 20% from the main analysis.
